# Inhibitory Effect of Depolymerized Sulfated Galactans from Marine Red Algae on the Growth and Adhesion of Diarrheagenic *Escherichia coli*

**DOI:** 10.3390/md17120694

**Published:** 2019-12-10

**Authors:** Yixiang Liu, Wenqiang Liu, Yanbo Wang, Yu Ma, Ling Huang, Chao Zou, Donghui Li, Min-Jie Cao, Guang-Ming Liu

**Affiliations:** 1 College of Food and Biological Engineering, Jimei University, Xiamen 361021, Chinamjcao@jmu.edu.cn (M.-J.C.); gmliu@jmu.edu.cn (G.-M.L.); 2 Xiamen Key Laboratory of Marine Functional Food, Xiamen 361021, China; 3 School of Food Science and Biotechnology, Zhejiang Gongshang University, Hangzhou 310018, China; wangyb@mail.zjgsu.edu.cn

**Keywords:** sulfated galactans, marine algae, enterotoxigenic *Escherichia coli*, anti-diarrhea, anti-adhesion

## Abstract

Active polysaccharides as safe and natural polymers against bacterial diarrhea have been reconsidered as an alternative to antibiotics. This work investigated the inhibiting effect of depolymerized sulfated galactans from *Eucheuma serra* and *Gracilaria verrucosa* on the growth and adhesion of diarrheagenic enterotoxigenic *Escherichia coli* (ETEC) K88. Results showed that the sulfated polysaccharides with molecular weight distribution ≤20.0 kDa exhibited antibacterial activity against ETEC K88. A structure–activity study revealed that the anti-ETEC K88 activity of sulfated polysaccharides is strictly determined by their molecular weight distribution, sulfate group content, and monosaccharide composition. In addition, the promoted nucleic acid release and the fluorescence quenching of membrane proteins were observed after the treatment with selected polysaccharides. Scanning electron microscopy further confirmed that the depolymerized sulfated galactans can effectively inhibit ETEC K88 adhesion. In conclusion, depolymerized sulfated galactans exhibited an inhibitory effect on the growth and adhesion of ETEC K88.

## 1. Introduction

Morbidity and mortality from human intestinal pathogens, including diarrheagenic *Escherichia coli* (*E. coli*), has become an enormous global burden [1]. Diarrheagenic bacteria can enter the gastrointestinal digestive system through food, adhere to and infect gastrointestinal epidermal cells, and thus cause vomiting, diarrhea, and even intestinal bleeding [2]. Various antibiotics have been widely used in the treatment of diarrhea, and the curative effect is definite. However, the use of these drugs can damage the growth of intestinal probiotics [3]. In addition, owing to the long-term use of antibiotics, genetic mutations and selective pressure have pushed enteric pathogenic bacteria to become resistant or multi-resistant to antibiotics [3]. Therefore, the development of novel compounds as alternative therapies or preventive agents has become increasingly important. 

As the main bioactive components in marine algae, sulfated polysaccharides, particularly their bacteriostatic or bactericidal activity, have attracted attention [4]. In brown algae, the sulfated polysaccharides from *Laminaria japonica* and *Sargassum swartzii* can inhibit Gram-negative and -positive bacteria including *E. coli*, *Vibrio cholera*, *Salmonella typhi*, and *Staphylococcus aureus* [5,6]. The sulfated polysaccharides from green algae (*Chaetomorpha aerea*) and microalgae (*Porphyridium cruentum*) also display inhibitory effect on *S. aureus* and *Salmonella enteritidis*, respectively [7,8]. For red algae, the sulfated polysaccharide from *Hypnea musciformis* can effectively inhibit diarrhea in rodents [9]. 

As the traditional edible red seaweed in China, *Eucheuma serra* and *Gracilari verrucosa* have become the main raw materials for the production of carrageenan and agar, respectively [10]. Compared with those from other algae, the sulfated polysaccharide from red algae is more homogeneous in sugar composition. These polysaccharides usually have about 90% of linear backbone built up of alternating 3-linked β-d-galactopyranose and 4-linked α-d-galactopyranose residues [11]. The hydroxyl groups of galactose residues in side chains may be substituted by ester sulfate and methyl groups [12]. Therefore, the major sulfated polysaccharides in red algae are generally in the form of sulfated galactans [13]. A recent study showed that the sulfated galactans from *Gracilaria ornate* exhibit antibacterial activity against only *E. coli* among two Gram-positive and five Gram-negative bacteria [14]. 

As Gram-negative bacteria, enterotoxigenic *E. coli* (ETEC) K88 is a worldwide cause of severe diarrhea in humans and animals [1]. In this study, two kinds of sulfated galactans were extracted from *E. serra* and *G. verrucosa*. The inhibitory effect of sulfated galactans on the growth of diarrheagenic ETEC K88 was investigated. In addition, the anti-adhesive property of sulfated galactans against ETEC K88 was discussed. This work is important in utilizing red algae to produce natural antibacterial polysaccharides. 

## 2. Results 

### 2.1. Yield and Composition of Polysaccharides

Sulfated polysaccharides were obtained through hot water extraction and ethanol precipitation. The yields of crude sulfated galactans extracted from *E. serra* and *G. verrucosa* were 19.5% (w/w) and 7.6% (w/w), respectively. The chemical compositions of the sulfated galactans are summarized in Table 1. *E. serra* sulfated polysaccharide (ESP) was composed of total carbohydrate 78.3%, sulfate 28.2%, 3,6-anhydrogalactose (3,6-AG) 9.8%, and uronic acid 2.2%. For its monosaccharide composition, galactose (93.4%) was the major component, and small amounts of glucose (0.6%), glucuronic acid (0.9%), galacturonic acid (0.9%), xylose (1.1%), and mannose (3.2%) were also found. *G. verrucosa* sulfated polysaccharide (GSP) has the similar monosaccharide composition except for the xylose content. The GSP was composed of total carbohydrate 83.8%, sulfate 13.1%, 3,6-AG 13.4%, and uronic acid 4.2%. ESP and GSP have mixed sugars, and their monosaccharide units are mainly galactose; however, ESP has higher sulfate content but lower uronic acid and 3,6-AG content than GSP. 

### 2.2. Effect of Depolymerization on the Antibacterial Activity of Sulfated Galactans

**Figure 1:**
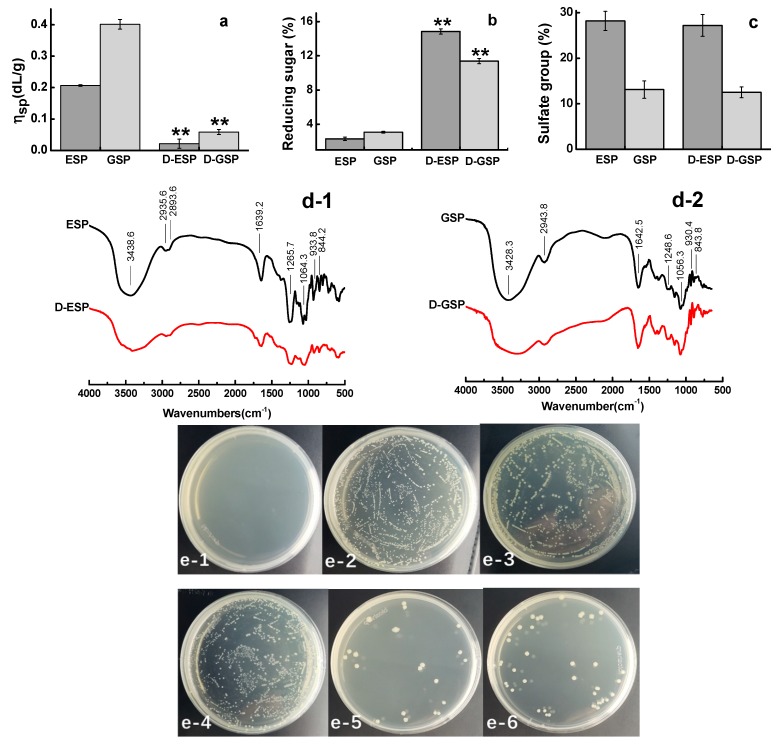
Effect of depolymerization on the antibacterial activity of sulfated galactans. (**a**) Viscosity, (**b**) reducing sugar, (**c**) sulfate group, (**d**) FT-IR analysis of *E. serra* sulfated polysaccharide (ESP) and depolymerized ESP (D-ESP) (**d-1**) and *G. verrucosa* sulfated polysaccharide (GSP) and depolymerized GSP (D-GSP) (**d-2**), and (**e**) antibacterial activity of sulfated galactans against enterotoxigenic *E. coli* (ETEC) K88: (**e-1**) positive control (0.05 mg/mL kanamycin), (**e-2**) negative control (0.85% NaCl saline), (**e-3**) 7.5 mg/mL ESP, (**e-4**) 7.5 mg/mL GSP, (**e-5**) 7.5 mg/mL D-ESP, and (**e-6**) 7.5 mg/mL D-GSP.

**Figure 2:**
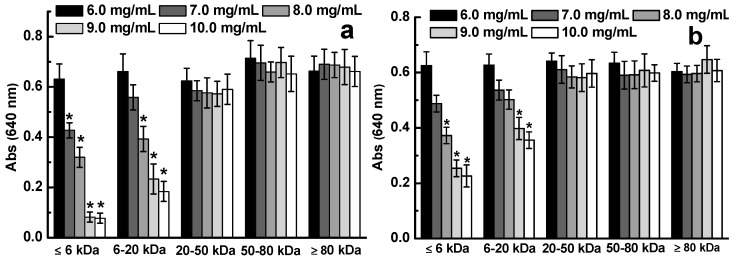
Effect of molecular weight on the antibacterial activity of D-ESP (**a**) and D-GSP (**b**).

**Figure 3:**
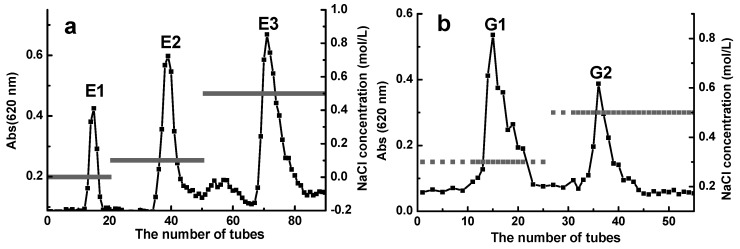
Separation of <6 kDa D-ESP (**a**) and D-GSP (**b**) by diethylaminoethyl (DEAE)-cellulose 52 anion exchange chromatography and comparative analysis of their antibacterial activity against ETEC-K88.

**Figure 4:**
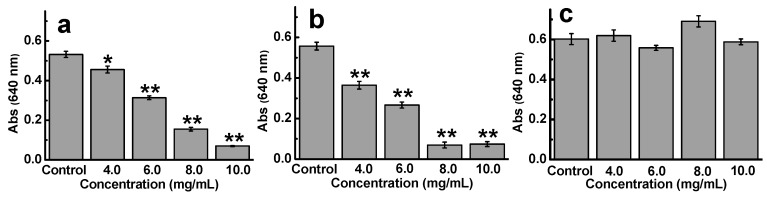
Effect of monosaccharide composition on the anti-ETEC K88 activity of sulfated polysaccharides. (**a**–**c**) are D-ESP (≤6 kDa, 23.8% sulfate), fucoidans from *Laminaria japonica* (≤6 kDa, 23.1% sulfate), and standard dextran sulfate (5 kDa, 20% sulfate), respectively.

**Figure 5:**
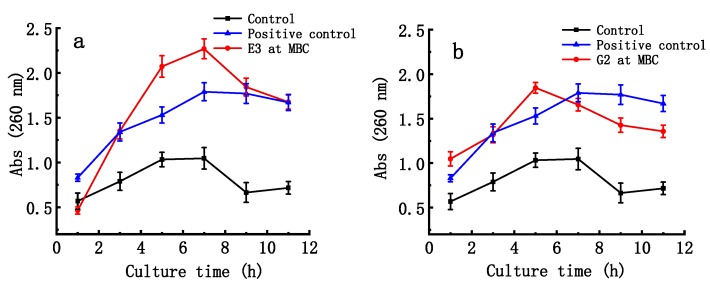
Effect of depolymerized sulfated galactans on nucleic acid releases in bacterial suspension at the minimal bactericidal concentration (MBC). (**a**,**b**) show nucleic release in time pattern after ETEC K88 samples were treated with the E3 fraction of D-ESP and G2 fraction of D-GSP, respectively. Positive control is kanamycin (0.05 mg/mL).

**Figure 6:**
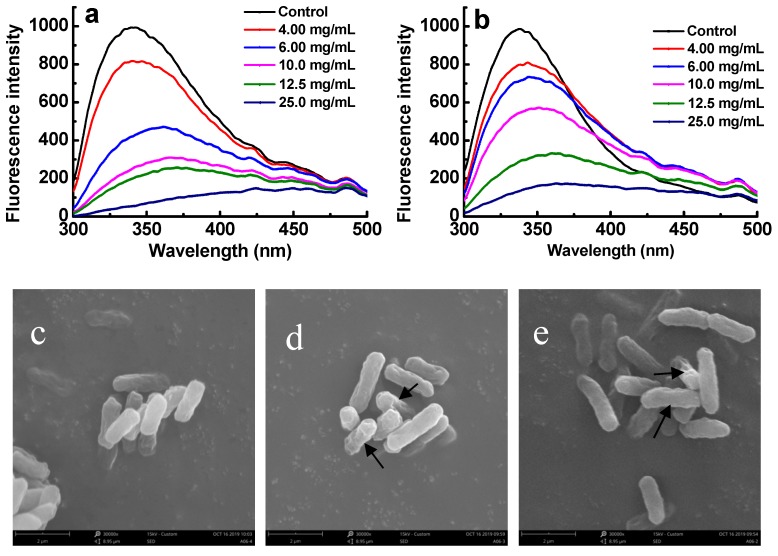
Effect of sulfated galactans on cell membrane of ETEC K88. (**a**,**b**) show fluorescence intensity changes of bacterial suspensions exposed to the E3 fraction of D-ESP and G2 fraction of D-GSP for 5 h. (**c**–**e**) respectively show the SEM images of ETEC K88 without and with E3 and G2 treatment under their MBC concentrations for 5 h.

After being hydrolyzed by high temperature and pressure, ESP and GSP had significantly decreased intrinsic viscosity (*p* < 0.01) and increased content of reducing sugar (*p* < 0.01) (Figure 1a,b). The sulfate contents of ESP and GSP were 28.2% and 13.1%, respectively, but no significant (*p* < 0.05) changes were observed in their depolymerized products (Figure 1c). The results of FT-IR spectrum analysis are shown in Figure 1d-1,2. The sulfated polysaccharides before and after depolymerization shared the similar spectral feature. ESP and GSP and their corresponding depolymerized products exhibited absorption peaks at 3438, 2935, and 1064 cm^−1^, which are characteristic absorptions of -OH, C-H, and C-O, respectively [15]. The peak at 933 cm^−1^ can be attributed to 3,6-AG (C-O-S) [16]. The most important peaks for ESP and GSP and their depolymerized products are located at approximately 1244 and 1265 cm^−1^, respectively, which correspond to the stretching vibration of the ester sulfate groups (S=O) [6,17]. Considering the stronger sulfated group peak in ESP spectra than that in GSP, the former was more sulfated than the latter. This result is consistent with those in Table 1. The effect of depolymerization on the antibacterial activity of sulfated galactans is shown in Figure 1e-1–6. Compared with the depolymerized polysaccharides, the unprocessed ones did not show antibacterial activity against ETEC K88. However, the growth of ETEC K88 was inhibited on the culture plates for D-ESP and D-GSP at 7.5 mg/mL.

### 2.3. Effect of Molecular Weight on Antibacterial Activity

D-ESP and D-GSP were divided into different fractions by using ultrafiltration membranes with different molecular interceptions to clarify the molecular weight distribution of the depolymerized sulfated galactans that can effectively inhibit diarrhea-causing ETEC K88. As shown in Figure 2, the ≤20 kDa fractions in D-ESP and D-GSP displayed varying degrees of antibacterial activity against ETEC K88 in a dose-dependent manner ranging from 6.0 mg/mL to 10.0 mg/mL. For D-ESP and D-GSP, the antibacterial activity of the ≤6 kDa fraction was better than that of the 6–20 kDa fraction. However, when the molecular weight of the sulfated galactans was >20 kDa, no antibacterial activity was observed. According to the results in Table 2, the minimal inhibitory concentration (MIC) values of 6–20 kDa and ≤6 kDa fractions in D-ESP were also lower than that of the corresponding two fractions in D-GSP. These data indicated that ≤20 kDa sulfated galactans can effectively inhibit ETEC K88 growth. As shown in Table 2, the active products account for 66.7% in D-ESP and 55.7% in D-GSP.

According to ours and other previous studies, sulfate groups of marine polysaccharides are closely related with their biological activity, such as antibacterial and anti-tumor activities [3,6]. The content of sulfate groups should be another important factor for the inhibiting effect of sulfated galactans on ETEC K88. Compared with those of D-GSP, the ≤6 kDa and 6–20 kDa fractions of D-ESP exhibited higher inhibiting activity for ETEC K88 growth (Figure 2). As shown in Table 2, the sulfate group content was 27.2% and 31.4% for the ≤6 kDa and 6–20 kDa fractions in D-ESP, respectively, but was only about 12% for the two corresponding fractions in D-GSP. It is indicated that there may be a correlation between sulfate group content and antibacteral activity for sulfated galactans against ETEC K88. In the following experiments, the influence of anionic properties on the anti-ETEC K88 activity of depolymerized sulfated galactans was further discussed.

### 2.4. Effect of Anionic Properties on Antibacterial Activity

The ≤6 kDa D-ESP and D-GSP were further separated into different fractions by using an anion exchange chromatograph to illustrate the effect of polyanionic property on the anti-ETEC K88 activity of depolymerized sulfated galactans. As shown in Figure 3a,b, three fractions E1, E2, and E3 were obtained from D-ESP by sequentially eluting it with 0, 0.1, and 0.5 mol/L NaCl. For D-GSP, G1 and G2 fractions were eluted by 0.3 and 0.5 mol/L NaCl, respectively. Table 3 shows the MIC and minimal bactericidal concentration (MBC) values of different polysaccharide fractions with different sulfate group and uronic acid contents in the sugar chain. Owing to the low uronic acid content, the sulfate group may be the main factor contributing to the polyanionic characteristics of the investigated polysaccharides. However, this hypothesis needs further verification. As shown in Table 3, the E3 fraction with 8.0 mg/mL MIC and 12.5 mg/mL MBC displayed better anti-ETEC K88 activity and had higher sulfate group content compared with other fractions.

### 2.5. Effect of Monosaccharide Composition on Antibacterial Activity 

To reveal the effect of monosaccharide composition on the anti-ETEC K88 activity of sulfated galactans, D-ESP, fucoidan, and dextran sulfate were selected in this work. These polysaccharides have similar sulfate content (23.8%, 23.1%, and 20.0% for D-ESP, fucoidan, and dextran sulfate, respectively) and molecular weight distribution (≤6 kDa for D-ESP and fucoidan and 5 kDa for dextran sulfate) but different monosaccharide composition. Figure 4a–c show the anti-ETEC K88 activity of these three polysaccharides. Compared with D-ESP, fucodian ranging from 4.0 mg/mL to 8.0 mg/mL displayed better inhibitory effect on ETEC K88 growth. These results were confirmed by the MIC values, in which the ≤6 kDa fractions for D-ESP and fucoidan were 15.0 and 12.5 mg/mL, respectively. However, no anti-ETEC K88 activity was observed for dextran sulfate even at 10.0 mg/mL. Therefore, how monosaccharide composition affects the antibacterial function of polysaccharides remains to be further studied.

### 2.6. Effect of Sulfated Galactans on Cell Membrane Integrity

As shown in Figure 5, the absorbance of the suspensions of ETEC K88 at 260 nm significantly increased with time after the bacteria were treated with the E3 fraction of D-ESP and the G2 fraction of D-GSP at their MBC concentrations (*p* < 0.01). After 5 h, maximum absorption was reached for both fractions. From 5 h to 11 h, the nucleic acid release of ETEC-K88 treated with E3 fraction was significantly higher than that of ETEC K88 treated with G2 fraction (*p* < 0.05). This result indicates that the E3 fraction is more destructive to bacterial cell membrane than the G2 fraction. 

### 2.7. Effect of Sulfated Galactans on Cell Membrane Proteins

Attributing to the residues, including tryptophan, tyrosine, and phenylalanine, proteins usually can exhibit fluorescence property [6]. Therefore, if the membrane proteins of bacteria were combined with sulfated polysaccharides, their fluorescence properties should be suppressed [6]. The changes of membrane proteins were investigated to reveal the antibacterial mechanisms of sulfated galactans against ETEC-K88. Figure 6a,b shows that after ETEC K88 was exposed to E3 and G2 fractions for 4 h, a dose-dependent decrease in fluorescence intensity was observed. When the concentration of sulfated galactans was increased to 6.0 mg/mL, the maximal fluorescence intensity decreased from 1000 to 480 and 710 for E3 and G2 fractions, respectively. This result implied that the E3 fraction was more easily bound to the cell surface of ETEC-K88 than the G2 fraction. A red shift of the maximum emission wavelength was also observed. In addition, after being administered with the E3 and G2 fractions under the MBC concentrations for 5 h, the morphological and physical changes of treated bacteria were observed by SEM (Figure 6c–e). These images suggested that the depolymerized sulfated galactans might destroy the surface of the tested bacteria. The surfaces of the treated ETEC K88 underwent obvious morphological changes compared with the untreated control. Untreated cells were regular, intact, and with smooth surfaces (Figure 6c), whereas the bacterial cells treated with the E3 and G2 fractions became deformed, pitted, and shriveled (Figure 6d,e).

### 2.8. Effect of Sulfated Galactans on Bacterial Adhesion

**Figure 7:**
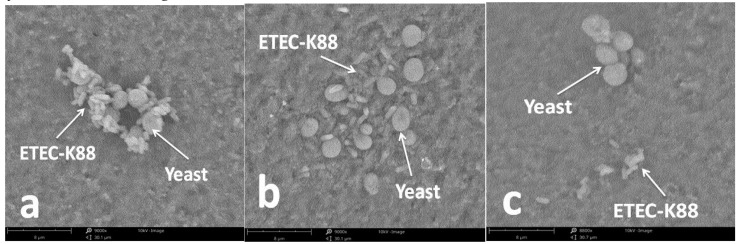
Effect of sulfated galactans on the adhesion of ETEC K88 on the surface of yeast cells observed by SEM after 4 h treatment. (**a**) is control (no sulfated galactans), and (**b**,**c**) represent E3 and G2 fractions both at 6.0 mg/mL, respectively.

When *S. cerevisiae* or ETEC K88 was exposed to 6.0 mg/mL of E3 or G2 fraction for 4 h, no obvious changes of microbial viability were observed. Therefore, under these conditions, the effect of sulfated galactans on bacterial adhesion was investigated. Figure 7a shows that after 4 h of co-incubation, a certain number of ETEC K88 accumulated around the cell wall of *S. cerevisiae*. However, the aggregation state of *S. cerevisiae* and ETEC K88 was ameliorated when E3 and G2 fractions were added to the culture system at 6.0 mg/mL. According to the SEM observations in Figure 7b,c, ETEC K88 did not adhere on the cell wall of *S. cerevisiae* after the treatment of depolymerized sulfated galactans.

## 3. Discussion

In this work, two kinds of sulfated galactans were extracted from *E. serra* and *G. verrucosa*. Although their monosaccharide units are mainly galactose, they differ in sulfate content, uronic acid, and 3,6-AG content. Several factors, including environmental conditions, seasonal variation, physiological factors, and extraction methods contribute to the variation in sulfated galactan yield from red seaweeds [18]. The chemical and structural properties of sulfated galactans in red algae vary within species. Even in the same species, the polysaccharide showed structural differences depending on life cycle stage and geographical location [9]. For example, the sulfated galactans extracted from the red seaweed *Mastocarpus stellatus* are composed of 95.2% galactose and minor amounts of xylose (2.5%) and glucose (2.4%) [18], whereas those from *Gracilaria genus* are composed mainly of alternating d-galactose and l-galactose [11].

Interestingly, only depolymerized sulfated galactans displayed anti-ETEC K88 activity according to our present results. For D-ESP and D-GSP, the antibacterial activity of the ≤6 kDa fraction was better than that of the 6–20 kDa fraction. However, when the molecular weight of the sulfated galactans was >20 kDa, no antibacterial activity was observed. This result is consistent with our earlier findings, in which the ≤6 kDa fucoidan shows the highest antibacterial activity [6]. It is believed that sulfated polysaccharides (such as fucoidans) with low molecular weight can easily penetrate cell membranes [19,20]. Thus, molecular weight should be one of critical factors determining the antibacterial activity of sulfated polysaccharides. However, the cell structure of Gram-negative bacteria is different from that of animal cells. Gram-negative bacteria, such as *E. coli*, have an outer membrane. Therefore, whether sulfated galactans can penetrate cell membrane of *E. coli* remains to be further studied.

Sulfated polysaccharides usually exhibit polyanionic property due to their abundant contents of sulfate group and uronic acid [6]. Binding and reacting with the glycoprotein-receptors on the bacterial cell surface are suggested as the antibacterial mechanisms of polysaccharides [21]. Therefore, the charge property of sulfated galactans may affect its interaction with bacteria. According to present research, the E3 fraction with 8.0 mg/mL MIC and 12.5 mg/mL MBC displayed better anti-ETEC K88 activity and had higher sulfate group content compared with other fractions. These results are consistent with earlier reports, in which the sulfated polysaccharides from *Pleurotus eryngii* with 0.69 degree of sulfonation exhibited better inhibition against *E. coli* and *S. aureus* than those from *Streptococcus thermophilus* ASCC 1275 with 0.31 degree of sulfonation [22]. Owing to the low uronic acid content, the sulfate group should be the main factor contributing to the polyanionic characteristics of the investigated polysaccharides. Therefore, the polyanionic property endowed by sulfate groups could be an effective modification to improve the anti-ETEC K88 activity of sulfated galactans.

In addition to molecular weight, charge density, and sulfate content, the monosaccharide unit plays an important role in the biological actions of polysaccharides [21]. In present research, no anti-ETEC K88 activity was observed for dextran sulfate even at 10.0 mg/mL. This result is consistent with our earlier research, in which dextran sulfate even exhibited a promoting role in the proliferation of nonpathogenic *E. coli* [6]. According to our present results, the monosaccharide units of D-ESP are composed of 93.4% galactose, 3.2% mannose, and small amounts of xylose, glucuronic acid, and galacturonic acid. According to our earlier studies, the selected fucoidan is composed of 47.2% fuctose, 26.6% mannose, 15.0% glucose, and 11.2% galactose [6]. However, the monosaccharide composition of dextran sulfate is 100% glucose. As the pathway of bacteria invading host cells, some specific proteins (polysaccharide receptors) on the surface of microorganisms can specifically bind to the heparin sulfate on the surface of host cells [23]. Gut et al. discovered mannose-specific lectins on the fimbriae of Enterobacteria (e.g., *E. coli* or *S. aureus*) that bind to human intestinal cells [24]. The immunomodulating activity of mushroom polysaccharides containing glucose and mannose should be attributed to their binding ability with the glucose/mannose-specific receptors on human macrophages [25,26]. Therefore, the antimicrobial activity of sulfated polysaccharides might be achieved by the specific recognizing and binding of polysaccharides receptors on the bacterial surface. These actions may be regulated by the monosaccharide composition of polysaccharides. We discovered that after the administration of depolymerized sulfated galactans, the fluorescence intensity of membrane protein can be suppressed and the surfaces of ETEC K88 became deformed, pitted, and shriveled. As far as the dextran sulfate, based on above discussion, it is reasonable to believe that although dextran sulfate could bind to the polysaccharide receptor on the bacterial surface, it cannot effectively inhibit bacterial growth. This hypothesis was supported by previous studies, in which *Cordyceps cicadae* polysaccharide was also believed to serve as nutrition for *E*· *coli* growth after 24 h cultivation [27,28]. However, according to recent studies, it is reported that sulfated galactans are required for growth of Gram-positive *Micrococcus luteus* [29]. Therefore, the effects of sulfated polysaccharide on different microorganisms remain to be further studied. 

After binding to the bacterial surface, anti-bacterial polysaccharides can further destroy the membrane structure of bacteria [30]. The increased absorbance of bacterial suspension at 260 nm is due to the leakage of intracellular nucleic acids; this phenomenon is a good indicator for evaluating the integrity of cell membranes [31]. In present work, significant nucleic acid release of ETEC K88 was observed after being exposed to depolymerized sulfated galactans. This result is consistent with earlier reports, in which the sulfated polysaccharides from *Laminaria japonica* and *Sargassum polycystum* also display a destructive effect on the membrane permeability for Gram-negative and -positive bacteria, including *E. coli*, *S. aureus*, and *Pseudomonas aeruginosa* [6,21]. However, at longer times, the amount of 260 nm absorbing material tended to decrease with time. This may be attributed to the adsorption of the 260 nm absorbing material on the precipitates during the precipitation process, which were filtered out prior to the OD measurement [32].

The interaction of specific proteins on the surface of microorganisms (adhesins) with carbohydrate chains on the glycoconjugate (receptors) of host cells enables the microbes to take their first step toward establishing an infection [24]. Therefore, inhibiting the adhesion and colonization of diarrheal pathogens on intestinal epithelium is an important therapeutic part of effectively fighting bacterial diarrhea. Gram-negative enteropathogenic bacteria including *E. coli* exhibit a strong binding ability to *Saccharomyces cerevisiae* strains [33,34]. The coagulation reaction between yeast and Gram-negative bacteria can be used as a model to simulate and study the adhesion of pathogenic bacteria to host mammalian cells [34]. Sulfated polysaccharides (e.g., heparan sulfate and chondroitin sulfate) are present on animal cells and act as the receptors of microbial adhesions [23]. Owing to their similar structure and charge property with the receptors of microbial adhesins on the host cell surface, sulfated polysaccharides may competitively bind with pathogenic bacteria and thus effectively inhibit the invasion of pathogenic bacteria on the host cells. According to our present results, the depolymerized sulfated galactans from *G. verrucosa* and *E. serra* exhibited an effectiveness in inhibiting the adhesion of diarrheogenic ETEC K88. Therefore, marine sulfated galactans have potential to replace antibiotic drugs in the treatment of bacterial diarrhea.

## 4. Materials and Methods 

### 4.1. Chemicalsand Reagents

Dextran sulfate and monosaccharide standards including xylose, mannose, galactose, glucose, glucuronic acid, and galacturonic acid were purchased from Sigma Chemical Co. (St. Louis, MO, USA). DEAE-Cellulose used for separating depolymerized sulfated polysaccharides was obtained from Sigma (Sydney, Australia). Brain heart infusion (BHI) was purchased from Shanghai Gaochuang Chemical Technology Ltd. (Shanghai, China). Deionized water was produced using a Milli-Q unit (Milipore, Bedford, MA, USA). Other analytical reagent-grade reagents were purchased from China National Pharmaceutical Industry Corporation Ltd. (Shanghai, China). The dinitrosalicylic acid (DNS) reagent was prepared according to the following steps: 10 g DNS and 300 g of sodium potassium tartrate was added to 800 mL of 0.5 mol/L NaOH aqueous solution and was gently heated to dissolve the reagents, and then the volume was made up to 1.0 L with distilled water. 

### 4.2. Extraction of Crude Sulfated Galactans

The cultivated fresh *E. serra* (from Qingdao in China) and *G. verrucosa* (from Putian in China) were respectively collected in May and March 2017. The sample was washed three times with tap water to remove salt, epiphytes, and sand attached to the surface. The seaweeds were then dried, ground into powder (40 mesh sieved) and stored at 4 °C until use. The sulfated polysaccharide was isolated from *E. serra* according to a previous study [15], with some modifications. Briefly, 100 g of the dried algae powder was macerated in hot water extraction at 55 °C for 4 h. The *E. serra* syrup was then filtered through filter cloth, concentrated to 1/4th of the original volume, cooled, and precipitated with three volumes of ethanol overnight at 4 °C. The precipitate was collected by centrifugation and washed 3 times with 75% ethanol, dehydrated and lyophilized to get a dried crude *E. serra* sulfated polysaccharide (ESP).

The extraction of the sulfated polysaccharide from the marine algae *G. verrucosa* was performed as described previously [35], with some modifications. Briefly, the *G. verrucosa* powder was extracted twice with hot water with a mass volume ratio of 1:40 (g/mL) at 55 °C for 4 h and filtered through the filter cloth. The combined extracts were concentrated to 1/4th of initial volume in a rotary evaporator under reduced pressure at 55 °C. Then 95% (v/v) ethanol was added to the concentrated supernatants with constant stirring to achieve a final concentration of 40% ethanol. The solution was left at 4 °C overnight, centrifuged at 5000 r/min for 10 min. The supernatant was added 95% ethanol again to a final concentration of 80% ethanol and kept at 4 °C overnight. The polysaccharides were collected by centrifugation as above and washed 3 times with 75% ethanol, dehydrated and lyophilized to get a dried crude *G. verrucosa* sulfated polysaccharide (GSP).

### 4.3. Purification of Sulfated Galactans

The crude sulfated polysaccharides were dissolved in distilled water, and the small molecules, especially for mineral substances were removed by Millipore Labscale TFF System as described in our previous studies [6]. Then, the concentrated polysaccharide solution underwent deproteinization according to the Sevag method [6]. The polysaccharide solution and the Sevag reagent (chloroform/*n*-butanol 4:1, v/v) were mixed (polysaccharide solution/Sevag reagent 5:1, v/v), thoroughly shaken for 30 min, centrifuged to remove the denatured proteins, and repeated five times. After the precipitate was collected and lyophilized, the polysaccharide powder was kept in a glass desiccator at room temperature until use.

### 4.4. Analysis of Chemical Composition 

Total carbohydrate content was determined by the anthrone-sulfuric acid test using galactose as the standard [36]. Sulfate content was determined by BaCl_2_ turbidimetric method [37]. Uronic acid content was determined by the modified sulfuric acid-carbazole method using glucuronic acid as standard (Sigma) [38]. The 3,6-anhydrogalactose (3,6-AG) was measured by the ester sulfate-methoxyl method using fructose as standard [39].

The monosaccharide compositions of sulfated polysaccharides were analyzed by high performance liquid charomatography (HPLC) according to a previous report [12]. Briefly, ten milligram samples were firstly hydrolyzed with 2 mol/L trifluoroacetic acid (TFA) aqueous solution in sealed ampoule bottles at 120 °C for 6 h in an oven. The hydrolyzates were removed through decompression and distillation with methanol for three times. Subsequently, the above monosaccharide mixtures and monosaccharide standers were derived into 1-phenyl-3-methyl-5-pyrazolone (PMP) methanol solution and extracted for the HPLC analysis. The samples were filtered through 0.22 μm syringe filter and injected in an Agilent1100 HPLC system with an Atlantis C18 column (4.6 × 250 mm, 5 μm) and a refractive index (RI) detector. The following monosaccharide standers were used as references: xylose, mannose galactose, glucose, glucuronic acid, and galacturonic acid.

### 4.5. Fourier Transform Infrared Spectroscopy (FT-IR) Analysis

The structural characteristics of the sulfated polysaccharides were determined by FT-IR spectroscopy [15]. Briefly, 5 mg of sample was milled in a mortar with 100 mg of dried spectrotometer grade potassium bromide (KBr) powder and then pressed into pellets in preparing as salt disc (10 mm/dm) for FT-IR measurement in the frequency range of 500–4000 cm^−1^ at a resolution of 4 cm^−1^.

### 4.6. Preparation of Depolymerized Sulfated Galactans and Physicochemical Property Analysis

#### 4.6.1. Depolymerization and Molecular Weight Classification

Preparation of low molecular weight polysaccharides was according to previous studies [6]. Polysaccharides containing 30% (w/w) deionized water were degraded using an autoclave reactor (Shanghai Boxun Industry & Commerce Co. Ltd. Medical Equipment Factory, Shanghai, China) at 121 °C and 0.103 MPa for 40 min. Subsequently, the depolymerized productions were dissolved in deionized water and were separated further by an ultrafiltration device (Millipore). Then, five fractions with different molecular weight regions (F1, ≤6 kDa, F2, 6–20 kDa; F3, 20–50 kDa; F4, 50–80 kDa; F5, >80 kDa) were obtained. These fractions were lyophilized and kept in a glass desiccator at room temperature until use.

#### 4.6.2. Reducing Sugars and Viscosity

The reducing sugars content was determined using the DNS assay [40]. An aliquot of each sample (150 μL, 1.0 mg/mL) was mixed with 150 μL of the DNS reagent in a test tube and the mixture was incubated in a boiling water bath for 5 min. After cooling to room temperature, the absorbance of the supernatant was measured at 540 nm. A calibration curve was prepared using galacturonic acid (0–0.2 mg/mL).

The viscometer of sulfated galactans depolymerized before and after was determined using a size 75 Ubbelohde semi-micro dilution type capillary viscometer (Hangzhou Zhuoxiang Technology Co. Ltd., Hangzhou, China) at 25 °C [41]. Briefly, the viscometer and polysaccharides solutions were kept in a temperature controlled water bath at 25 °C. The concentration of polysaccharide solution was set as 5 mg/mL, and the elapsed time that different samples going through the Ubbelohde viscometer was recorded. The intrinsic viscosity [*η*] value was obtained by plotting [*η*_sv_/*C*] against *C* using the Huggins equation and the *C* was extrapolated to zero [42].

*η*_sv_/*C *= [*η*] + *κ*[*η*]^2^*C* (1)

where *C* is the concentration of the sample (g/mL), *κ* is the Huggins constant, *η*_sv_ is the specific viscosity of the sample.

### 4.7. Fractionation by Ion-Exchange Chromatography

The depolymerized sulfated galactans were dissolved in distilled water at 5.0 mg/mL and then fractionated using a 50 cm × 2.6 cm i.d. glass column filled with DEAE-Cellulose 52 resin. After balancing by deionized water, a linear gradient of NaCl solution (0 to 0.5 mol/L) was used to elute different polysaccharide fractions at a flow rate of 1.0 mL/min. The collected fractions were determined for the carbohydrate content by anthrone-sulfuric acid method, and the fractions showing higher carbohydrate yield were pooled together. The collected fractions were lyophilized and kept in a glass desiccators at room temperature until use.

### 4.8. Microbial Strains and Culture

Standard strain of Gram-negative enterotoxigenic *E. coli* (ETEC K88) (CN-3-321) was purchased from Beijing Biobw Biotechnology Co., Ltd. (Biobw, Beijing, China). The bacteria were grown in Luria-Bertani (LB) medium and incubated at 37 °C with shaking (180 rpm/min) for 12 h. Cultures were harvested by centrifugation at 5000× *g* for 10 min, and the pellet was washed twice and re-suspended in sterile phosphate buffer saline (PBS) to form about 10^6^ colony forming units (CFU) mL^−1^.

The wild *Saccharomyces cerevisiae* strain BY4741 was provided by Euroscarf (Oberursel, Germany). The yeast strains were maintained in medium containing 1% yeast extract, 2% peptone and 25% glycerol and stored at −80 °C. For all experiments, yeast cells were grown in YPG medium (1% yeast extract, 2% peptone, and 2% glucose) at 28 °C for 24 h under constant agitation (150 rpm/min). Then, the yeasts were harvested by centrifugation at 7000× *g* for 10 min and re-suspended in PBS at a concentration of 10^8^ CFU/mL.

### 4.9. Antibacterial Assay

The antibacterial activity of the sulfated polysaccharides was determined using both the liquid turbidity method and plate smearing method [43,44]. The ETEC K88 in the logarithmic growth period was centrifuged to remove the supernatant and then diluted to a concentration of 10^6^ CFU/mL by normal saline water. In each tube, 1.6 mL LB medium, 2.0 mL polysaccharide solution, and 400 μL ETEC K88 suspension were mixed. The final concentrations of polysaccharides were 2.0, 4.0, 6.0, 8.0, and 10 mg/mL, respectively. After incubation at 37 °C for 24 h, the absorbance was read at 640 nm with a microplate reader (TECAN infinite 200pro, Tecan AG, Switzerland).

The susceptibility of ETEC K88 to the sulfated galactans was also validated using the plate smearing method. Firstly, the polysaccharide aqueous solutions were added into the sterilized nutrient agar before solidification, and the final concentration of polysaccharides was 7.5 mg/mL. After cooling, 100 μL ETEC K88 suspension (10^6^ CFU/mL) was spread on the surface of nutrient agar containing sulfated galactans. After incubation at 37 °C for 24 h, the number of colonies on the plate surfaces was counted. Sterilized water was used as negative control, and kanamycin (0.05 mg/mL) was used as positive control.

### 4.10. Minimal Inhibitory Concentration (MIC) and Minimal Bactericidal Concentration (MBC)

The MIC and MBC of sulfated galactans were carried out as described by the National Committee for Clinical Laboratory Standards [45], with slight modification. The MIC was determined as the lowest concentration of polysaccharides at which no bacterial growth was detected after incubating for 24 h, whereas the MBC was the lowest concentration of the test polysaccharides that showed no growth in the culture after incubating at 37 °C for 48 h [6]. The polysaccharide was dissolved in sterilized 0.85% NaCl saline with a concentration of 50 mg/mL. The serial dilutions of the polysaccharide (25.0, 12.5, 10.0, 8.0, 6.25, 3.13, 1.56 mg/mL) were prepared for both MIC and MBC tests. The tests were carried out in LB culture with an inoculum about 10^6^ CFU/mL. A series of tube dilutions were incubated on a rotary shaker at 180 rpm/min for 24 h or 48 h at 37 °C. Kanamycin solution (0.05 mg/mL) was used as positive controls, and the sterilized 0.85% NaCl saline was used as the negative control. 

### 4.11. Integrity of Cell Membrane

The integrity of bacterial cell membrane was examined according to the release of nucleic acid into supernatant [45]. The ETEC K88 was grown overnight at 37 °C in BHI, and the microorganisms were collected by centrifugation at 5000 rpm for 15 min. Then, bacteria were washed trice and re-suspended in pH 7.4 PBS. The OD_640_ of the suspension was adjusted to 2.0 ± 0.02. Subsequently, after being filtered with sterilized 0.02 μm filter membranes, the bacterium removal polysaccharides with MBC concentration were added into the bacterial suspensions. Then, the bacterial suspensions were incubated at 37 °C under agitation (150 rpm/min). The samples were centrifuged at 11,000 g for 5 min and the supernatants were measured by an ultraviolet spectrophotometer (UV-755B) at 260 nm.

### 4.12. Effect on Cell Membrane Proteins

The binding effect of the sulfated galactans on bacterial membrane proteins was investigated by using an immunofluorescence method according to previous studies [6]. The ETEC K88 was cultured overnight at 37 °C in BHI. Bacteria were harvested by centrifugation at 8000 rpm for 10 min. The precipitated cells were washed thrice and re-suspended with sterilized PBS (pH 7.4). Then, the OD_640_ of bacterial suspension was adjusted to 1.2 ± 0.02. Subsequently, after being filtered with sterilized 0.02 μm filter membranes, the polysaccharides were added into the bacterial suspensions, and the final concentrations were 4.0, 6.0, 10.0, 12.5, and 25.0 mg/mL. Then, the suspensions were incubated at 37 °C for 5 h, and the fluorescence level of cells was scanned ranging from 280 nm to 500 nm using a fluorescence spectrophotomemter (Cary Eclipse) at 340 nm excitation. The light slit width for both excitation and emission was 5 nm.

### 4.13. Inhibition of Bacterial Adhesion

Adhesion of bacteria onto yeast cells was performed as previously described [46], with some modification. Briefly, the cultured yeast and ETEC K88 in suspensions was harvested by centrifugation at 8000 rpm/min for 10 min, respectively. The precipitated cells were washed thrice and re-suspended with sterilized PBS (pH7.4). In the sterile centrifugal tube, 1.0 mL (10^8^ CFU/mL) yeast suspension, 0.5 mL (10^9^ CFU/mL) ETEC K88 suspension, and 0.5 mL PBS containing 6.0 mg/mL sulfated galactans were vortexed for 1.0 min and incubated at 37 °C for 4 h. 

To morphologically observe the bacterial adhesion, scanning electron microscope (SEM) observation was performed according to a previous report [47]. The yeast-bacteria suspension was fixed overnight with 2.5% (v/v) glutaraldehyde in PBS solution (pH 7.4) at room temperature. Then, the fixed bacterial pellets were submitted to dehydration with increasing ethanol concentrations (30% to 100%), and then the ethanol was replaced with tertiary butyl alcohol. Subsequently, cells were dried after centrifugation at “critical point” in liquid CO_2_ under 95 bar pressure, and samples were covered in gold by cathodic spraying. Finally, the samples were observed on a SEM (PHENOMWORLD PW-100-016, Netherlands).

### 4.14. Statistical Analysis

The statistical significance of the differences between the control and treatment groups was determined by one-way ANOVA using Origin version 8.0, followed by Tukey tests. A normality test showed that all the raw data had a normal distribution, and all groups were determined to have equal variance by a variance test. Data were expressed as mean ± SD of at least three individual experiments, each run in triplicate. *p* < 0.05 (two-sided) was considered significant (*, *p* < 0.05; **, *p* < 0.01).

## 5. Conclusions

Two kinds of sulfated galactans (ESP and GSP) were obtained from *E. serra* and *G. verrucosa*, respectively, to explore the application of sulfated polysaccharides from marine red alga in fighting against bacterial diarrhea. Their structure–activity relationship and mechanisms in inhibiting the growth and adhesion of diarrhea-causing ETEC K88 were also investigated. After being depolymerized, the ≤6 kDa sulfated galactans exhibited the best antibacterial activity. However, no activity was observed when their molecular weight was >20 kDa. In addition, their polyanionic property and monosaccharide composition were confirmed to influence their inhibitory activity against ETEC K88. On the aspect of antibacterial mechanism, the depolymerized sulfated galactans can bind to the membrane proteins and destroy the membrane systems of ETEC K88, ultimately leading to bacterial cell death. These depolymerized sulfated galactans can also effectively inhibit the adhesion of ETEC K88 on yeast cells, indicating their potential function in preventing the invasion of diarrheagenic bacteria on intestinal epithelium. In summary, this study emphasized the important role of depolymerized sulfated galactans from *E. serra* and *G. verrucosa* as a promising source for natural anti-diarrhea agents.

## Figures and Tables

**Table 1:** Yield and chemical composition of crude polysaccharide from red seaweeds.

Items		ESP ^a^	GSP ^b^
Yield (%, w/w) ^c^		19.5 ± 1.2	7.6 ± 0.5
Sulfate (%, w/w)		28.2 ± 2.1	13.1 ± 1.9
Total carbohydrate (%, w/w)		78.3 ± 4.5	83.8 ± 3.1
Uronic acid (%, w/w)		2.2 ± 0.3	4.2 ± 0.4
3,6-anhydrogalactose (%, w/w)		9.8 ± 0.3	13.4 ± 0.5
Monosaccharide composition (%) ^d^	Mannose	3.2	2.7
	Glucuronic acid	0.9	0.4
	Galacturonic acid	0.9	1.2
	Glucose	0.6	2.2
	Galactose	93.4	93.5
	Xylose	1.1	0

^a^ Sulfated polysaccharide extracted from *Eucheuma serra*. ^b^ Sulfate polysaccharide extracted from *Gracilaria verrucosa*. ^c^ Calculated according to the dry weight of algae. ^d^ Calculated according to the peak area ratio of the detected monosaccharides in liquid chromatogram.

**Table 2:** Yields and sulfate group contents of different fractions of D-ESP and D-GSP.

Fractions	D-ESP			D-GSP		
	Yield (%)	Sulfate Group (%)	MIC ^a^ (mg/mL)	Yield (%)	Sulfate Group (%)	MIC (mg/mL)
Mixure	100.0	28.3 ± 1.9	25.0	100.0	13.3 ± 1.7	40.0
≤6 kDa	43.2	27.2 ± 2.4	15.0	22.6	12.5 ± 1.2	25.0
6–20 kDa	23.5	31.4 ± 3.4	20.0	33.1	12.1 ± 2.1	30.0
20–50 kDa	16.9	29.7 ± 2.6	-	7.5	10.5 ± 2.3	-
50–80 kDa	9.3	31.4 ± 3.2	-	9.3	12.4 ± 2.4	-
>80 kDa	7.1	24.9 ± 1.9	-	27.5	11.6 ± 1.1	-

^a^ minimal inhibitory concentration. “-“ means no antimicrobial activity.

**Table 3:** Antibacterial activity of sulfate polysaccharides with different anionic properties.

Test Items	D-ESP			D-GSP	
	E1	E2	E3	G1	G2
MIC (mg/mL)	10.0	10.0	8.0	12.5	10.0
MBC (mg/mL)	25	25	12.5	25.0	25.0
Sulfate group (%, w/w)	19.4 ± 2.8	23.8 ± 3.1	29.2 ± 2.2	16.9 ± 2.1	23.1 ± 1.4
Uronic acid (%, w/w)	0.7 ± 0.1	1.2 ± 0.1	1.6 ± 0.1	2.2 ± 0.2	2.9 ± 0.2
